# Characterization of Pilin A and Thioredoxin A Mutant Strains of *Acinetobacter baumannii*, From a Transposon Insertion Library, for Pili Production and Virulence‐Associated Properties

**DOI:** 10.1002/mbo3.70183

**Published:** 2025-11-27

**Authors:** Jadelynn Aki, Sara B. Papp, Bayley Polk, Sean Jeffreys, Megan P. Tompkins, Anwar A. Kalalah, Mark Eppinger, Guoquan Zhang, M. N. Guentzel, James P. Chambers, Bernard P. Arulanandam, Jieh‐Juen Yu

**Affiliations:** ^1^ Department of Molecular Microbiology and Immunology University of Texas at San Antonio San Antonio Texas USA; ^2^ Department of Immunology Tufts University School of Medicine Boston Massachusetts USA

**Keywords:** *Acinetobacter baumannii*, pilin, pilus, thioredoxin, virulence

## Abstract

*Acinetobacter baumannii* is a multi‐drug resistant Gram‐negative coccobacillus. It is responsible for high mortality among patients in the intensive care unit. Reported *A. baumannii* virulence factors include the thioredoxin system which plays a critical role in gene regulation and protein reduction. The Type IV pilus (T4P) is a well‐known bacterial virulence factor that is associated with adhesion and molecular exchange. Previously, our laboratory revealed the role of *A. baumannii* thioredoxin A (TrxA) in pathogenesis by studying a *trxA* deletion mutant that downregulates T4P gene expression. TrxA, a potent disulfide bond reducer, might affect the assembly of pili by targeting T4P component proteins, including PilA, the major pilin protein of T4P which contains multiple cysteine residues required for disulfide bond formation. Using a transposon library derived from the AB5075 clinical isolate, we phenotypically characterized a *pilA* mutant strain and compared its pathogenesis to the wild type (WT) strain as well as another *trxA* mutant. Whole genome sequencing was conducted to confirm the disruption of *trxA* and *pilA* genes in the corresponding mutant strains of AB5075. Alteration of bacterial surface appendages in Δ*trxA* and Δ*pilA* was visualized by Scanning electron microscopy. Like Δ*trxA*, the T4P mutant Δ*pilA* had marked reduction of surface pili. Bacterial attachment to excised intestinal surfaces was greatly reduced for Δ*trxA* and Δ*pilA*. Attenuation of Δ*trxA* and Δ*pilA* in pathogenesis was further confirmed using a mouse sepsis model. Collectively, this characterized Δ*pilA* deficiency in *A. baumannii* resulted in attenuation of virulence making it a potential therapeutic target.

## Graphical abstract

*Acinetobacter baumannii* transposon insertion mutant strains Δ*pilA* and Δ*trxA* display marked reductions of surface pili. The PilA (a major pilin protein) and TrxA (thioredoxin A) deficient bacteria also show attenuation in pathogenesis including the reduction of intestinal bacterial attachment, increased phagocytic uptake and decreased mortality from systemic infection.

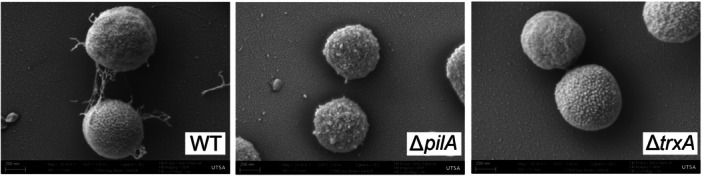

## 1 Introduction

*Acinetobacter baumannii* is one of the major causes of healthcare‐associated infections (Moghadam et al. 2018). The ability to survive by avoiding desiccation and death on surfaces in the hospital environment, and the increase in multi‐drug resistance (MDR) make it a member of the ESKAPE nosocomial pathogens (Nordmann 2004). Ventilator‐associated pneumonia and catheter‐related bloodstream/urinary tract infections are the main manifestations of *A. baumannii* infection (Joly‐Guillou 2005; O'Shea 2012; Morris et al. 2019). Additionally, *A. baumannii* is one of the leading organisms observed with bacterial co‐infection and superinfection with SARS‐CoV‐2 due to extended hospitalization of COVID‐19 patients (Musuuza et al. 2021; Rangel et al. 2021). Empirical treatment for *A. baumannii* is generally ineffective due to its MDR nature, resulting in severe outcomes (Zilberberg et al. 2016). The pathogenesis and virulence factors contributing to infectivity of *A. baumannii* have yet to be fully understood. Studies to identify virulence factors that influence *Acinetobacter* pathogenicity have been conducted using various approaches including phenotypic characterization, genomic manipulation and infection model analyses. These studies have led to the discovery of many *Acinetobacter* virulence factors including secretion systems, efflux systems, outer membrane vesicles, capsule, nutrient acquisition systems, and proteins involved in community interactions and virulence gene regulation (Morris et al. 2019; Harding et al. 2018; Zhou et al. 2023).

By studying differences in *A. baumannii* colonization in the gut of wild‐type and IgA deficient mice, we determined bacterial thioredoxin A (TrxA) acts as a pathogenesis‐associated protein by dissociating secretory component from sIgA (Ketter et al. 2018). TrxA is a small ubiquitous redox protein that plays an important role in the cellular response to oxidative stress. It is also involved in transcription regulation, protein folding and degradation, chaperon activity, and several biosynthetic pathways (Kumar et al. 2004). Our study using an *A. baumannii trxA* gene deletion strain (Δ*trxA*) derived from the clinical isolate Ci79 further revealed the regulatory role of TrxA in type IV pilus (T4P) biogenesis, first by transcriptomic analysis, and subsequently confirmed by Transmission electron microscopy (TEM) (May et al. 2019a). We found that many T4P component genes were downregulated and formation of surface pili was markedly reduced in Δ*trxA* (May et al. 2019a). How TrxA regulates T4P assembly is unknown. However, Reardon‐Robinson et al. reported that deletion of MdbA (containing a conserved thioredoxin‐like domain) prevented the assembly of shaft pilin SpaA in the Gram‐positive bacterium *Corynebacterium diphtheriae*, possibly by reducing the SpaA disulfide bond (Reardon‐Robinson et al. 2015). Potential TrxA target proteins within the *A. baumannii* T4P include PilA (a putative structural pilin) which contains disulfide‐bond forming cysteine residues. Without TrxA, PilA may not fold properly to form pili. We hypothesize that as the downstream target of TrxA, the PilA impaired mutants should share similarity in morphology, functional phenotype and virulence with Δ*trxA*. Due to limited marker gene selection for manipulation of multi‐drug resistant *A. baumannii*, we proceeded to test our hypothesis using Δ*trxA* and Δ*pilA* mutant strains from an AB5075 transposon insertion library generated in the Manoil Laboratory (Gallagher et al. 2015) of the University of Washington. SEM was used to visualize bacterial surface structures. Bacterial attachment to intestinal mucosal surfaces and susceptibility of mice to systemic *A. baumannii* infection were used to assess bacterial virulence.

## 2 Materials and Methods

### 2.1 Bacteria

*Acinetobacter baumannii* wild type (WT; AB5075‐UW) and mutant strains Δ*pilA* (tnab1_kr121127p04q131) and Δ*trxA* (tnab1_kr121210p04q139) in the *A. baumannii* AB5075 transposon mutant library (Gallagher et al. 2015) were purchased from The University of Washington, Seattle, WA, USA. WT AB5075 was grown in Millers LB medium supplemented with ampicillin (100 µg/mL) while Δ*trxA* and Δ*pilA* were grown in the same medium but supplemented with tetracycline (10 µg/mL).

### 2.2 Animals

Mouse experiments utilized 6‐week‐old female BALB/c mice purchased from Jackson Laboratory (Bar Harbor, ME, USA). Mice were housed at The University of Texas at San Antonio AAALAC‐accredited animal facility. All experiments were performed in accordance with an approved protocol, following guidelines set forth by the Institutional Animal Care and Use Committee (IACUC).

### 2.3 Genome Sequencing and Assembly

Total genomic DNA was extracted from AB5075 WT, Δ*pilA*, and Δ*trxA* strains using the GeneJET Genomic DNA Purification Kit (Thermo Fisher Scientific, Waltham, MA, USA), followed by short‐read sequencing on Illumina MiSeq. 2500 platform (San Diego, CA, USA) at the Tufts University Genomics Core Facility. Paired‐end libraries were prepared with the Illumina Nextera DNA XT (Illumina, San Diego, CA, USA) with 250‐bp read length and sequenced using the MiSeq Reagent kit (v.2) (500‐cycle). Sequencing reads in the fastq format were imported and processed in Galaxy (Afgan et al. 2022). Default software parameters were used for the below analyses, unless specified otherwise. Sequence read quality was assessed using FastQC (v.0.74+Galaxy0) (http://www.bioinformatics.babraham.ac.uk/projects/fastqc). Reads were assembled with SPAdes (v.3.15.4+Galaxy1) (Prjibelski et al. 2020), and resulting contigs evaluated with QUAST (v.5.2.0+Galaxy1) (Mikheenko et al. 2018), followed by annotation using the NCBI Prokaryotic Genome Annotation Pipeline (PGAP) (v.6.6) (Tatusova et al. 2016). Mutations relative to the closed reference chromosome of *A. baumannii* strain AB5057 (Jacobs et al. 2014) (GenBank CP113078.1) were catalogued using the breseq pipeline (Deatherage and Barrick 2014).

### 2.4 Scanning Electron Microscopy

*A. baumannii* AB5075 WT strain was grown overnight from a colony in 3 mL LB broth supplemented with ampicillin (100 µg/mL) at 37°C with shaking. The Δ*pilA* and Δ*trxA* mutants were grown under identical conditions but supplemented with 10 µg/mL tetracycline. Respective bacteria (150 µL) were sub‐cultured in 3 mL LB broth without antibiotics and grown at 37°C with shaking for 4 h to mid‐log growth phase. The culture growth at OD_600_ was adjusted to 0.5 with LB and 500 µL of each bacterial culture was added on top of a 5 × 5 mm silicon chip (Ted Pella Inc. #16008, Redding, CA, USA) in a 48 well plate. Samples were placed in a static incubator at 37°C for 48 h. Bacteria were washed 3 times with phosphate buffered saline (PBS, pH 7.4) and fixed with 0.25% glutaraldehyde (in PBS) at room temperature for 1 h. Specimens were dehydrated using 30% to 100% ethanol before critical point drying and sputter coating with gold (Ali et al. 2021). Samples were examined using a Zeiss Crossbeam 340 Focused Ion Beam Scanning Electron Microscope (Kleberg Advanced Microcopy Center, The University of Texas at San Antonio).

### 2.5 Motility Assay

Measurements of bacterial twitching motility were determined using a modified protocol reported by Wood et al (Wood et al. 2018). Briefly, overnight cultures of AB5075 WT, Δ*pilA*, and Δ*trxA* were normalized and collected using a PrecisionGlide Needle and stabbed through a 0.3% (w/v) agar LB layer to the agar/plate interface. Plates were grown right‐side up for 48 h. The soft agar was removed, and the diameter of bacterial growth was measured by staining with 0.1% (w/v) crystal violet.

### 2.6 Macrophage Uptake Assay

The RAW 264.7 macrophage cell line was cultured in D10 medium for 3 days. Cells were removed with trypsin and seeded in a 96‐well plate at a density of 10^5^ cells/well. The plate was incubated for 2 h at 37°C with 5% CO_2_ to allow for adherence. The media was aspirated and replaced with 200 µL diluted Interferon gamma (1 µg/mL), and the cells were incubated as described above overnight. *A. baumannii* AB5075 strain was grown overnight from a frozen stock in 3 mL LB broth supplemented with 100 µg/mL ampicillin at 37°C with shaking. Mutant strains Δ*pilA* and Δ*trxA* were grown under identical conditions but supplemented with 10 µg/mL tetracycline. Bacteria were sub‐cultured into fresh LB and grown at 37°C with shaking for 3 h. The OD_600_ for each strain was adjusted to approximately 0.3 and then diluted 1:10 in D10 medium. Serial dilutions of the bacterial suspensions were plated on LB agar and incubated at 37°C overnight for enumeration of input bacteria. Macrophage containing wells were washed twice with warmed antibiotic‐free D10 medium and then 100 µL of the respective 1:10 bacterial suspensions were added to the macrophages to achieve the desired multiplicity of infection (MOI) of 10. The plate was centrifuged at 300 xg for 2 min followed by incubation at 37°C in 5% CO_2_ for 1 h. Following incubation, the plate was transferred onto an ice pack in a 4°C refrigerator for 20 min. The media was aspirated and 200 µL polymyxin B (25 µg/mL) was added to each well and incubated at 4°C for 1 h. The plate was then washed twice with antibiotic‐free D10 medium and macrophages lysed with 200 µL 0.2% deoxycholate. Each well was serially diluted in PBS, plated on LB agar, and incubated at 37°C overnight. The percentage difference between the input bacteria and macrophage‐associated bacteria after lysing was calculated to determine macrophage uptake of bacteria.

### 2.7 Bacterial Intestinal Attachment Assay

Bacterial gut adhesion was investigated using a modified protocol reported by Ketter et al (Ketter et al. 2018). Briefly, bacteria were diluted to a concentration of 1 × 10^8^ CFU/mL. The small intestine obtained from a humanely euthanized female BALB/c mouse was cut into sections (approximately 2 cm in length). The intestinal lumen was exposed using fine scissors, and sections were placed in the bacterial suspension for 30 min with gentle agitation. Intestine sections were washed in sterile PBS (10 mL) by inverting seven times and straining through a cell strainer (45 µm) to remove unattached bacteria. The tissue was then suspended in 4 mL sterile PBS, soaked for 5 min, and strained through a cell strainer (45 µm). Washed tissue was then transferred to 2 mL sterile PBS and homogenized using a TissueMiser (Thermo Fisher, Waltham, MA, USA) for bacterial enumeration. Dilution plating was performed on LB agar containing 50 and 10 µg/mL chloramphenicol and cycloheximide, respectively, to inhibit growth of resident microbiota.

### 2.8 Acinetobacter Baumannii Intraperitoneal Challenge

Overnight cultures of AB5075 WT and mutants were subcultured and diluted to an OD_600_ of 0.03 in LB broth and grown for 4 h. Subcultures were then pelleted and washed with PBS. Each culture was diluted in PBS to 5 × 10^8^ CFU/mL. Each dilution was then diluted 10‐fold to reach the challenge concentration of 5 × 10^7^ CFU/mL. Actual bacterial concentrations were determined by plating serial dilutions on LB agar plates. BALB/c mice (*n* = 6 per group) were injected intraperitoneally with 100 µL of each respective inocula for a challenge dose of approximately 5 × 10^6^ CFU/mL. The challenged mice were monitored daily for morbidity and mortality for 20 days.

### 2.9 Statistical Analysis

Statistical Analyses were performed with Graphpad Prism 10.0. One‐way ANOVA followed by Tukey's multiple comparisons test or Kruskal‐Wallis test was used to compare the difference between two groups with normal or non‐normal sample distribution, respectively. Survival statistical differences between challenge groups were determined using the Mantel‐Cox log rank test. *P*‐values ≤ 0.05 are considered significant.

## 3 Results

### 3.1 Confirmation of Gene Disruption in ΔpilA and ΔtrxA Strains

To investigate the alteration of phenotype and associated gene deficient virulence, i.e, *pilA* and *trxA*, *A. baumannii* Δ*pilA* and Δ*trxA* strains from a AB5075 mutant library generated by T26 transposon random insertion (Gallagher et al. 2015), and their parental strain AB5075‐UW were procured from The University of Washington (USA). Whole bacterial genome sequencing confirmed that a single T26 transposon insertion in each of the Δ*pilA* and Δ*trxA* strains had disrupted *pilA* and *trxA* genes, respectively (Table 1). The sequence data sets have been deposited in the Sequence Read Archive (SRA) and GenBank at NCBI under BioProject PRJNA1003928.

**Table 1:** Whole genome sequencing of AB5075‐UW WT and mutant strains.

Strain	SRA accession	GenBank accession	T26 position^a^	Disrupted locus^a^	Putative disrupted gene function
WT	SRR25663643	GCA_031932345.1	**—**	**—**	
Δ*pilA*	SRR25663641	GCA_031932305.1	327955	ABUW_0304	Type IV pilin structural subunit
Δ*trxA*	SRR25663642	GCA_031932325.1	3309695	ABUW_3276	Thioredoxin

^a^ T26 transposon insertion site and disrupted locus mapping to AB5075‐UW complete genome (GenBank: NZ_CP008706.1).

**Figure 1:**
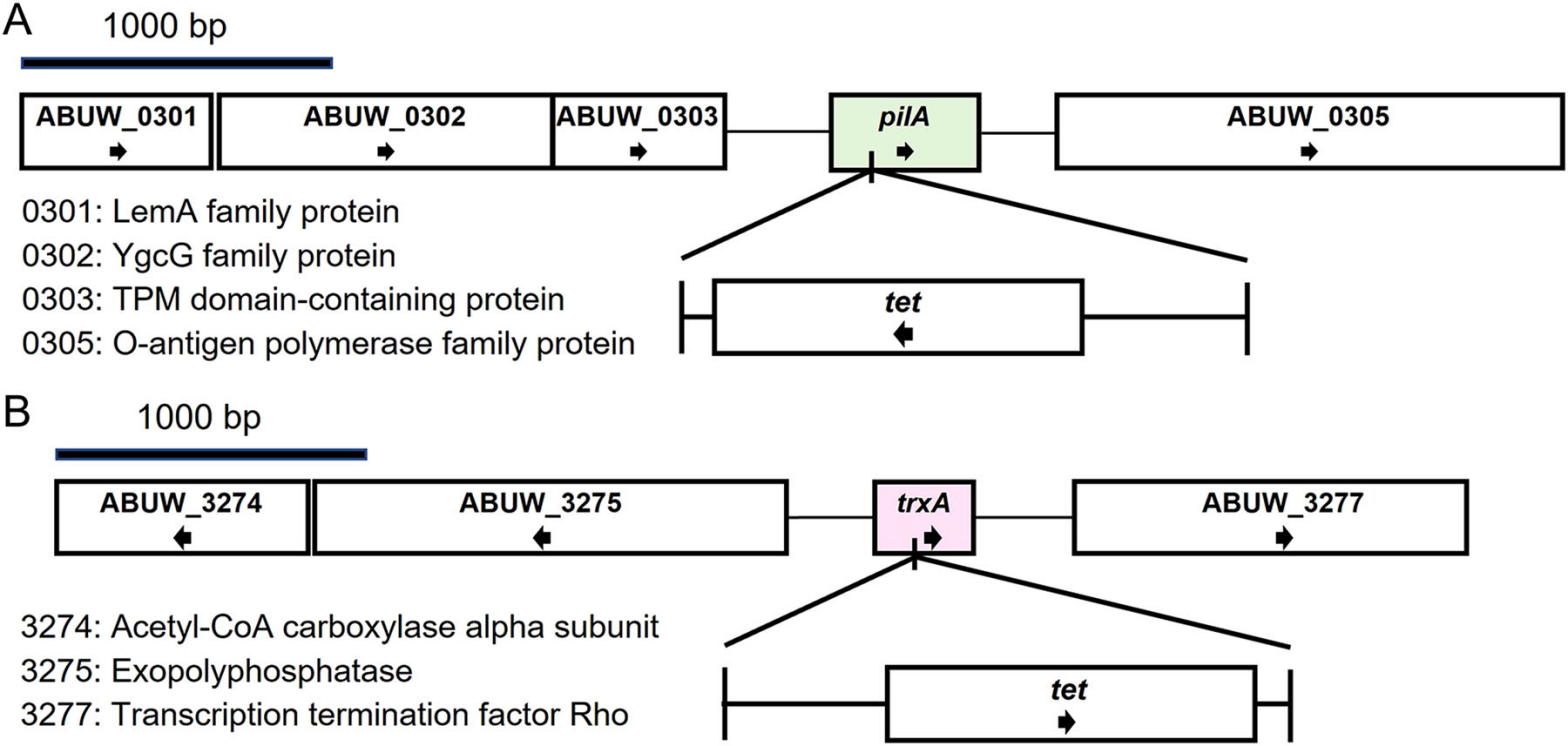
Disruption of AB5075‐UW *pilA* and *trxA* genes by T26 transposon insertion carrying a tetracycline efflux transporter gene *tet*. The illustration is based upon whole genome sequences of (A) Δ*pilA* (tnab1_kr121127p04q131) and (B) ΔtrxA (tnab1_kr121210p04q139) strains. Distance and putative encoding protein function of genes (immediate up‐ and down‐ stream) of the disrupted *pilA* and *trxA* genes are shown to support the unlikelihood of a polar effect due to gene disruption.

As shown in Figure 1A, the *pilA* gene is not associated with other T4P genes. The downstream ABUW_0305 encoding gene contains consensus sequences for both −10 and −35 elements at the promoter region for its gene transcription, thus disruption of *pilA* gene by T26 transposon carrying a tetracycline resistant gene is unlikely to have a polar effect on the expression of ABUW_0305. Similarly, the *trxA* gene is not part of an operon (Figure 1B).

### 3.2 ΔpilA and ΔTrxA Strains Are Deficient in Forming Surface Filaments

**Figure 2:**
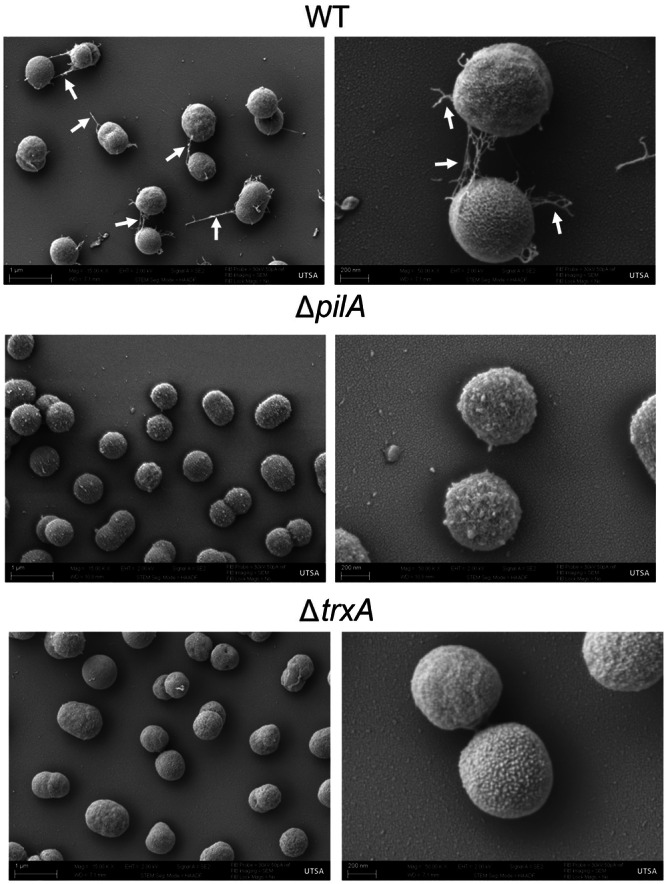
Scanning electron microscopy of *A. baumannii* AB5075‐UW wild type (WT) and mutant strains. Bacteria were grown statically on top of silicon chips for 48 h, fixed with 0.25% glutaraldehyde, critical point dried, sputter‐coated with gold, and visualized using a Zeiss Crossbeam 340 Focused Ion Beam Scanning Electron Microscope. Shown are representative micrographs for each respective strain of bacteria at 15000 and 50000 magnifications. A marked reduction of surface filaments (arrows) was evident in both Δ*pilA* and Δ*trxA* bacteria compared to the WT strain.

Gene sequence Blast analysis suggests that the AB5075‐UW *pilA* gene encodes Type IV major pilin. SEM assessment of 48 h static bacterial cultures revealed the formation of abundant pilus filaments of various lengths on the surface of WT AB5075‐UW while surface filaments were not visible in either the Δ*pilA* or Δ*trxA* strains (Figure 2).

### 3.3 Assessment of Bacterial Growth in Mutant Strains

**Figure 3:**
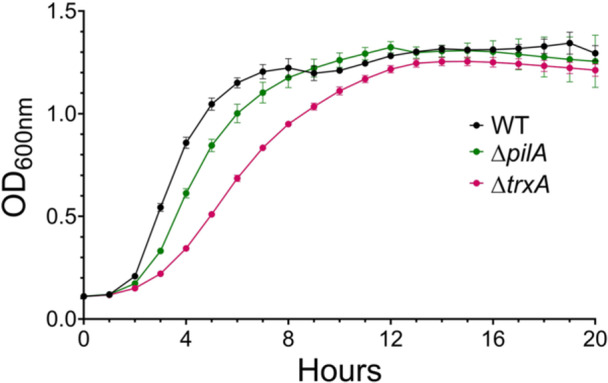
Comparison of growth characteristics of AB5075‐UW wild type (WT) and mutant strains. WT, Δ*pilA*, and Δ*trxA* bacteria were grown in Miller's LB broth at 37°C with shaking and OD_600_nm was determined hourly for 20 h.

The *in vitro* growth of WT and mutant strains in LB broth was measured hourly for 20 h with shaking (240 rpm) using a Spark multimode microplate reader (Tecan, Switzerland). All analyzed *Acinetobacter* strains displayed a typical growth cure with an initial adjustment lag phase, followed by an exponential (log) growth phase, and a stationary phase (Figure 3). The AB5075‐UW WT reached stationary phase at around 7 h while Δ*pilA* exhibited slightly delayed lag and log phase growth. In contrast, Δ*trxA* exhibited more prolonged lag and log phase growth when compared to WT. However, both mutant strains eventually reached stationary growth like that of the AB5075‐UW WT.

### 3.4 In Vitro Assessment of ΔpilA, and ΔtrxA Virulence Associated Phenotype

**Figure 4:**
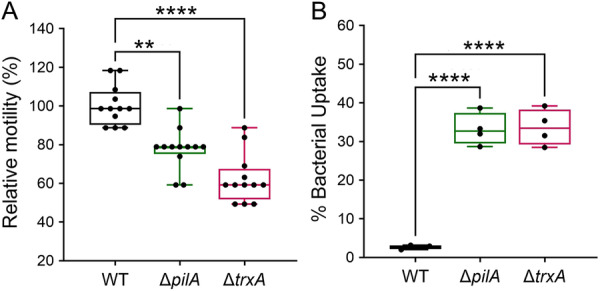
*In vitro* characteristics of AB5075‐UW wild type (WT) and mutant strains. (A) Relative motility of Δ*pilA*, and Δ*trxA* to WT strain (*n* = 12) was calculated by measurement of bacterial zone expansion at the agar‐plastic interphase. The box‐and‐whiskers graph indicates minimum, maximum, median, and 25th and 75th percentiles. **p = 0.0042, ****p < 0.0001, Kruskal‐Wallis test (one‐way ANOVA on ranks). (B) Bacterial uptake was measured by incubation of WT and mutants (*n* = 4) with RAW 264.7 cells at an MOI of 10 for 2 h (representative of two independent experiments). ****p < 0.0001, ordinary one‐way ANOVA followed by Tukey's multiple comparisons test.

*Acinetobacter* T4P has been shown to play a role in bacterial twitching motility (Harding et al. 2013). Deletion of the *trxA* gene in strain Ci79 also resulted in a significant reduction of twitching motility (May et al. 2019a). Since surface filaments were lacking in both AB5707 Δ*pilA* and Δ*trxA* mutant strains (Figure 1), we assessed whether twitching motility was impaired. Data from two independent experiments were combined (*n* = 12 total per group) and adjusted relative to the average WT motility zone designated as 100% to calculate relative motility for the mutant strains (Figure 4A). The results indicated an average of 22.3% and 37.6% motility reduction for Δ*pilA* and Δ*trxA*, respectively, compared to the WT strain. We further analyzed host immune cell responses to WT and mutants using a murine macrophage‐like RAW 264.7 cell line. The wild type AB5075 and mutant bacteria (Δ*pilA* and Δ*trxA*) were incubated with RAW 264.7 cells at an MOI of 10 for 2 h. Bacterial uptake by macrophages (Figure 4B) was significantly higher for Δ*pilA* (33.2 ± 2.1%) and Δ*trxA* (33.7 ± 2.3%) compared to AB5075 WT (2.7 ± 0.3%).

### 3.5 Assessment of Bacterial Attenuation in Pathogenesis

**Figure 5:**
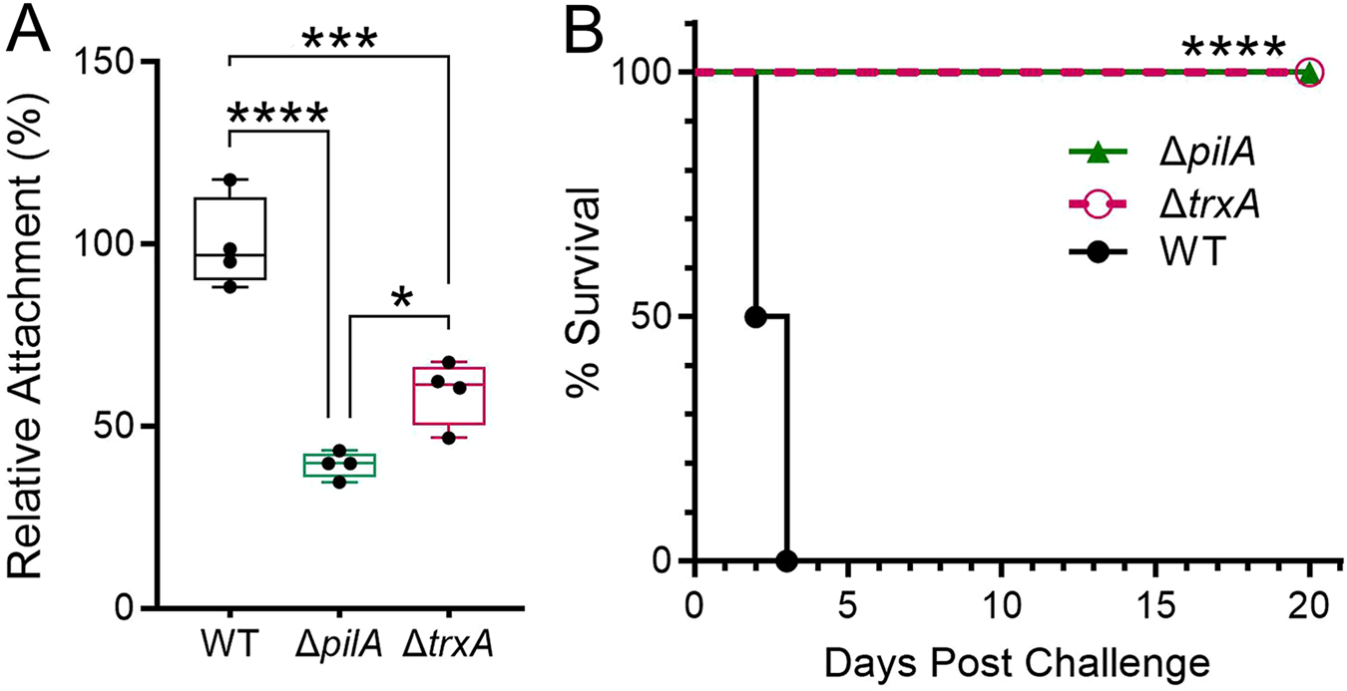
Comparison of pathogenesis of AB5075‐UW wild type (WT) and mutant strains. (A) BALB/c mice small intestine tissue was excised and cut into 2 cm lengths. The lumen was exposed and incubated with bacteria for 30 min. Attached bacteria were enumerated and relative attachment was calculated (*n* = 4 per group) using the average of WT numbers as 100%. The box‐and‐whiskers graph indicates minimum, maximum, median, and 25th and 75th percentiles. *p = 0.0317, ***p = 0.0004, ****p < 0.0001, one‐way ANOVA with Tukey's multiple comparisons test. (B) BALB/c mice (*n* = 6) were challenged with WT (4.3 ×10^6^ CFU), Δ*pilA* (5.0 ×10^6^ CFU), or Δ*trxA* (5.7 ×10^6^ CFU) via intraperitoneal injection. Survival of bacterium‐challenged mice was monitored for 20 days. ****p < 0.0001, Mantel‐Cox log rank test for each mutant compared to WT. Both animal experiments were conducted once to minimize animal usage.

Δ*pilA* and Δ*trxA* virulence attenuation was assessed using *ex vivo* intestinal bacterial attachment and *in vivo* intraperitoneal challenge assays. Bacteria were allowed to attach to excised small intestine segments with the mucosa exposed for 30 min. As shown in Figure 5A, relative intestinal attachment of Δ*pilA* (39.4 ± 1.8%) and Δ*trxA* (59.3 ± 4.4%) was greatly reduced when compared to AB5075‐UW WT (100 ± 6.3%). Bacterial pathogenesis was assessed using a systemic infection mouse model. BALB/c mice were challenged intraperitoneally with comparable, i.e., 4–6 x 10^6^ CFU of WT and respective mutant strains. All WT challenged mice succumbed to infection by day 3 post challenge, while all mice challenged with Δ*pilA* or Δ*trxA* survived the entire monitoring period of 20 days (experimental endpoint) suggesting the contribution of *pilA* and *trxA* genes and their encoding proteins to *A. baumannii* pathogenesis (Figure 5B).

## 4 Conclusions

In this study, we characterized a Δ*pilA* (tnab1_kr121127p04q131) mutant whose *pilA* gene was disrupted by a T26 transposon. PilA is the major pilin in the *A. baumannii* T4P secretion system (Piepenbrink et al. 2016). Widely present in prokaryotes, T4P has been shown to have diverse roles and functions, i.e., adherence, surface motility, biofilm formation, and DNA uptake (Singh et al. 2022; Berry and Pelicic 2015). Importantly, T4P is essential for virulence in many bacterial pathogens (Craig et al. 2004; Ouyang et al. 2024; Schmidt et al. 2022; Martini et al. 2021; Dos Santos Souza et al. 2020). *A. baumannii* strains have diverse PilA proteins with near homology in the N‐terminal hydrophobic α‐helix but divergency in the C‐terminal region leading to functional specialization (Piepenbrink et al. 2016; Ronish et al. 2019). The AB5075 PilA protein has been characterized by Ronish et al. by complementing an *A. baumannii* M2 strain *pilA* mutant with *pilA* ^AB5075^, showing its major role in twitching motility, but minor role in biofilm formation (Ronish et al. 2019). Here, we further demonstrate by SEM that AB5075 PilA is the major pilin and has an important role in intestinal attachment and virulence in systemic *Acinetobacter* infection. Interaction of T4P pilin with host proteins appears to be a common means for bacterial adherence and subsequent initiation and establishment of infection in the host. For example, Banerjee et al. demonstrated that Group B *Streptococcus* PilA (an ancillary pilin) binds to extracellular matrix collagen, which then engages α_2_β_1_ integrins of the brain endothelium promoting bacterial attachment while increasing blood‐brain barrier permeability for bacterial entry into the central nervous system (Banerjee et al. 2011). Conversely, binding of *Pseudomonas aeruginosa* to human buccal epithelial cells was mediated through the bacterial major pilins with host glycosphingolipid receptors (Lee et al. 1994). Deletion of the *pilA* gene usually leads to the attenuation of bacterial virulence. For example, deletion of *pilA* in *Burkholderia pseudomallei* decreased bacterial adherence to respiratory cell lines, and reduced the killing of BALB/c mice (Essex‐Lopresti et al. 2005). A *Haemophilus influenzae pilA* mutant displayed impairment of adherence to human bronchial epithelial cell lines and colonization in the chinchilla respiratory tract (Jurcisek et al. 2007). PilA is also required for full virulence of the highly virulent human pathogen *Francisella tularensis* (Forslund et al. 2010). Given the important role of pilins in bacterial pathogenesis, anti‐pilin‐based immunotherapy has been explored to combat bacterial infections. Vaccination with recombinant PilA protein has been shown to increase humoral immunity, thus leading to effective protection against *Pseudomonas aeruginosa* infection (Korpi et al. 2016). Vaccination with rPilA also was shown to reduce the host inflammatory response and provided 80% protection in a mouse model of intraperitoneal *Glaesserella parasuis* challenge (An et al. 2023).

The pathogenic role of PilA in *A. baumannii* postulated in our current study is based on a transposon insertion mutant. Whole genome sequencing of Δ*pilA* confirmed a single T26 insertion, and this gene disruption unlikely impacted downstream gene expression via a polar effect. Generation of complemented strains is complicated by the limited availability of antibiotic selection markers for a multidrug resistant *A. baumannii*, such as AB5075. However, the Δ*trxA* phenotypes i.e., lacking surface filaments, reduced bacterial motility, increased uptake by macrophages, and attenuation in virulence in mice reported here for AB5075 are almost identical to the *trxA* gene‐targeted deletion strain derived from the Ci79 clinical isolate (May et al. 2019a, 2019b). The AB5075 Δ*pilA* was generated by a single Tn26 insertion. It is very likely that the observed Δ*pilA* phenotype is PilA‐specific; however, generation of a complemented strain will ultimately confirm our finding and the role of PilA in *A. baumannii* pathogenesis. The *A. baumannii* PilA mediated adhesion mechanisms for tissue attachment remain to be elucidated. However, PilA‐based immunotherapy against emergent MDR *A. baumannii* infection is worthy of further investigation and application.

The thioredoxin system is ubiquitous in bacteria and plays a critical role in bacterial survival and virulence by maintaining the thiol‐disulfide balance and defending against oxidative stress. Thioredoxin reduces oxidized cysteine residues, thus preventing the accumulation of harmful oxidized proteins that could disrupt cellular functions (Collet and Messens 2010). Thioredoxin also has been reported to function as a transcriptional regulator in *F. tularensis* for modulating other oxidative stress response regulators to improve survival in host macrophages (Ma et al. 2022). Our laboratory has previously shown that *A. baumannii* TrxA plays roles both in secretory IgA reduction for bacterial pathogenesis (Ketter et al. 2018) and T4P modulation for bacterial virulence (May et al. 2019b). The ∆*trxA* mutant strain was shown to exhibit a loss of pili on the cell surface when compared to its parental *A. baumannii* Ci79 strain by TEM. In this study, we observed the same defect in pilus formation in the AB5075 ∆*trxA* strain using SEM. Importantly, the phenotype of the AB5075 transposon *trxA* mutant characterized in the current study shared high similarity (if not identical characteristics) to the Ci79 Δ*trxA* mutant generated by targeting deletion in the previous study, with restoration of the parental WT phenotype in its complemented mutant strain (May et al. 2019a). Additionally, the similarity in the current study between ∆*trxA* and ∆*pilA* strains in pilus formation, twitching motility, intestinal attachment, susceptibility to phagocytic uptake, and virulence in mouse sepsis, suggests PilA might be one of TrxA target proteins. However, it remains to be determined whether modulation of PilA by TrxA is mediated by indirect transcriptional regulation or direct protein‐protein interaction or both. A recent review paper by Akbarzadeh et al. highlighted the latest and most popular technologies to study protein‐protein interactions (Akbarzadeh et al. 2024). We will apply the well‐established *in vitro* pull‐down assay and the *in vivo* Yeast 2 Hybrid system to investigate whether TrxA interacts with PilA directly. Transcriptional regulation of *pilA* by TrxA will be assessed in a future study using qRT‐PCR to compare the difference in *pilA* gene expression between WT and ∆*trxA* AB5075 strains.

## Author Contributions

**Jadelynn Aki:** investigation. **Sara B. Papp:** investigation. **Bayley Polk:** investigation. **Sean Jeffreys:** investigation. **Megan P. Tompkins:** investigation. **Anwar A. Kalalah:** data curation, formal analysis. **Mark Eppinger:** validation. **Guoquan Zhang:** methodology. **M. N. Guentzel:** validation, writing – review and editing. **James P. Chambers:** writing – review and editing. **Bernard P. Arulanandam:** conceptualization, funding acquisition, supervision. **Jieh‐Juen Yu:** conceptualization, methodology, formal analysis, funding acquisition, resources, supervision, writing – original draft.

## Ethics Statement

The authors have nothing to report.

## Conflicts of Interest

The authors declare no conflict of interest.

## Data Availability

The datasets generated and/or analyzed during the current study are available in the GenBank at NCBI under BioProject PRJNA1003928 (https://www.ncbi.nlm.nih.gov/bioproject/PRJNA1003928/).
